# Recent applications of plant cell culture technology in cosmetics and foods

**DOI:** 10.1002/elsc.202000078

**Published:** 2020-12-18

**Authors:** Gergana Krasteva, Vasil Georgiev, Atanas Pavlov

**Affiliations:** ^1^ Laboratory of Cell Biosystems The Stephan Angeloff Institute of Microbiology Bulgarian Academy of Sciences Plovdiv Bulgaria; ^2^ Department of Analytical Chemistry and Physicochemistry University of Food Technologies Plovdiv Bulgaria

**Keywords:** active substances, adventitious roots, cosmeceuticals, food additives, plant cell suspension

## Abstract

Plants have been used as the main source of phytochemicals with nutritional, medicinal, cultural and cosmetic applications since times immemorial. Nowadays, achieving sustainable development, global climate change, restricted access to fresh water, limited food supply and growing energy demands are among the critical global challenges faced by humanity. Plant cell culture technology has the potential to address some of these challenges by providing effective tools for sustainable supply of phyto‐ingredients with reduced energy, carbon and water footprints. The main aim of this review is to discuss the recent trends in the development of plant cell culture technologies for production of plant‐derived substances with application in food products and cosmetic formulations. The specific technological steps and requirements for the final products are discussed in the light of the advances in cultivation technologies used for growing differentiated and undifferentiated plant in vitro systems. Future prospects and existing challenges of the commercialization of plant cell culture‐derived products have been outlined through the prism of the authors’ point of view. We expect this review will encourage scientists, policymakers and business enterprises to join efforts for speeding‐up the mass commercialization and popularization of plant cell culture technology as an eco‐friendly alternative method for sustainable production of plant‐derived additives with application in food and cosmetic products.

## 1 INTRODUCTION

Plant cell cultures are capable of synthesizing a large array of phytochemicals which are used as pharmaceuticals, food additives and cosmetic ingredients. Although most researches have focused on the production of pharmaceutically important compounds, over the past decade there has been a remarkable advance in the development of plant cell culture technologies used for production of active cosmetic ingredients or food additives [1, 2, 3]. Large‐scale cultivation of undifferentiated and differentiated plant cell cultures is linked to several difficulties of technological, economic and legislative nature. However, in vitro growth of plant cells, tissue and organ cultures under a controlled environment is the most promising eco‐friendly technology for sustainable supply of valuable phyto‐ingredients even by rare, endemic, protected, threatened or endangered plant species. Plant cell culture technology has the potential to meet the continuously growing demand for bioactive natural compounds in the near future. Currently, when choosing their foods and cosmetics, most people prefer to use natural products with low ecological footprints. In line with this tendency, the last few years have seen an exponential increase in the number of cosmetics with active substances obtained by plant cell culture technology [3, 4]. The intensive search for innovations and development of new products with multiple specific activities are the driving forces behind the rapid progress in the development of plant cell culture‐derived active ingredients for the needs of the cosmetics industry [4]. With their multiple biological activities, plant cell culture extracts are extremely desirable due to the fact that they are a unique mix of secondary and primary metabolites occurring naturally in plant cells [1]. Moreover, the final cosmetic formulations usually contain active ingredients in minor concentrations, which allows the production of plant cell extracts in small volumes at reasonable prices that cover production costs. However, a look at the food market reveals that there are very few examples of plant cell culture‐derived products. In general, the food industry operates with large volumes of raw materials, hence large‐scale production of plant cells is mandatory to respond to this demand. In this case, the desired price of the final product, which should be as low as possible, rarely covers the production, labor and technological equipment costs. For example, some of the more optimistic pilot products, such as Cocovanol™ by DianaPlantSciences (the first plant cell culture‐derived food additive granted with GRAS approval status) and PhytoVanilla™ by ESCA genetics corporation (the first patented biotechnology for production of natural vanilla flavor by using plant cell culture technology) are no longer on the market.

The aim of this review is to summarize the recent trends in the developments of plant cell culture technologies for production of plant‐derived substances with application in foods and cosmetic formulations with a special focus on the latest innovations in the field.

## 2 CULTIVATION OF PLANT CELL, TISSUE AND ORGAN CULTURES WITH EMPHASIS ON THE PRODUCTION OF ACTIVE INGREDIENTS FOR FOODS AND COSMETICS

Cultivation of plant cell, tissue and organ cultures in a controlled aseptic environment is a well known and widely exploited technology for production of biologically active plant‐derived secondary metabolites for the needs of pharmacy [5, 6, 7]. Even though the titer of biologically active secondary metabolites is normally low in plant cells, they can be engineered to increase yields or target compounds [2, 5]. Moreover, plants readily undergo genetic transformation, which could grant them the ability to produce heterologous proteins of pharmaceutical importance [8, 9]. As a result, the strategy for “molecular farming” has been employed for mass production of various pharmaceuticals by using genetically modified plants and plant in vitro systems [9, 10, 11]. For example, the first plant‐made antibodies tested in a clinical trial were commercialized by Planet Biotechnology Inc (https://www.planetbiotechnology.com/index.html) [11]. The company used transiently and stably transformed tobacco plants to produce DPP4‐Fc, an immunoadhesin for the treatment of MERS‐CoV coronavirus infection and CMG2‐Fc (PBI‐220), an immunoadhesin for the treatment and prevention of inhalational anthrax. Another example is the production of Elelyso^®^ (taliglucerase alfa) in transgenic carrot cell suspension by Protalix BioTherapeutics, Inc. (http://protalix.com/technology/procellex-platform/) [10]. Elelyso^®^ is the first biotherapeutic expressed in plant cell suspension, which was approved by regulatory authorities around the world for long‐term enzyme replacement therapy for patients with a confirmed diagnosis of type 1 Gaucher disease.

**FIGURE 1:**
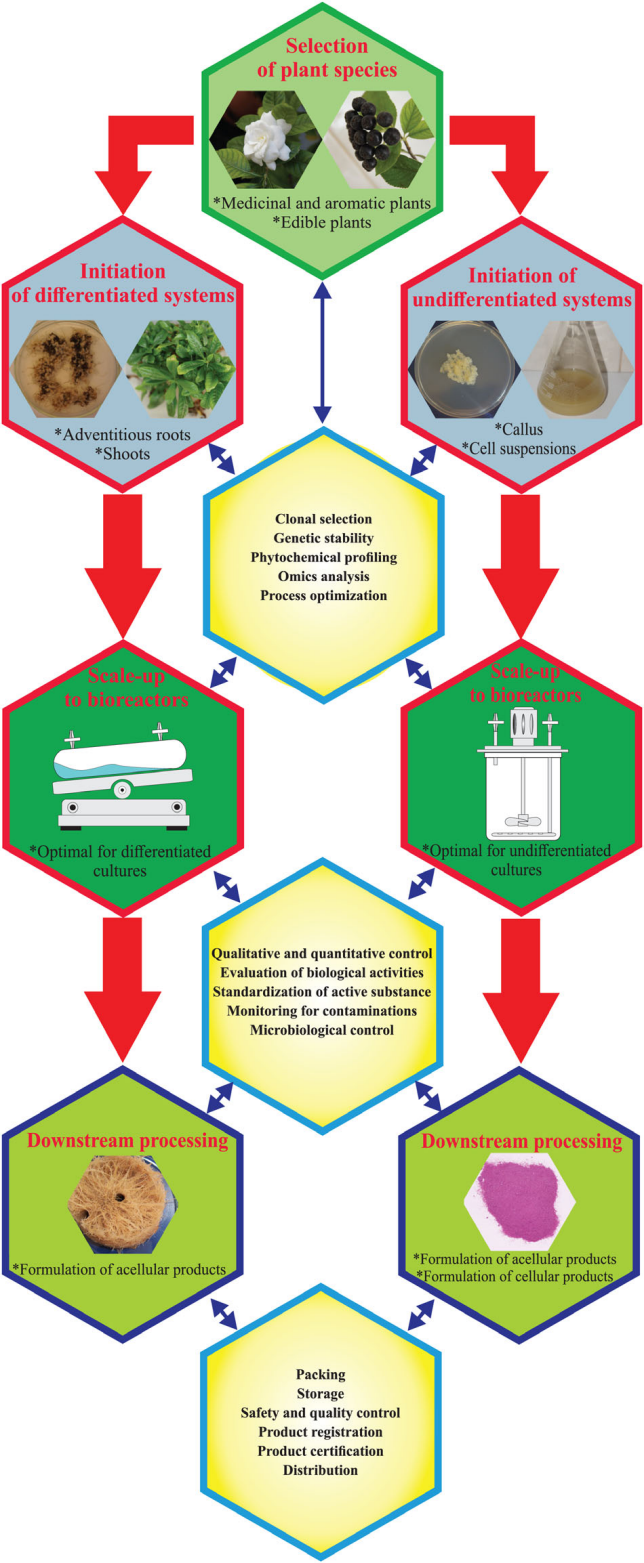
General steps in development of plant cell culture based technologies for production of foods and cosmetic ingredients

However, in contrast to the expression platforms used for the production of biotherapeutics for human pharmaceutical use, plant cell, tissue and organ cultures used for production of foods and cosmetic ingredients should not be genetically modified, since consumers are very sensitive to the presence of GMO. Following the modern perception of the concept of healthy lifestyle, the foods and cosmetics of the highest quality should meet the requirements for natural, GMO‐free, bio‐, vegan, eco‐friendly, and organic certificates. Moreover, the plant species used for initiation of in vitro cultures should belong to the edible or medicinal and aromatic plants, preferably with affirmed GRAS (generally recognized as safe) status. The plant cell culture technologies used for production of foods and cosmetic ingredients follow the same principles, with the only differences found in the working volumes and downstream processing of culture liquids [1]. Depending on the specific demands, the food industry requires much bigger amounts of final products than the cosmetic industry, where the plant cell culture‐derived products are usually added in minimal concentrations and serve as active ingredients [1]. It is obvious that plant cells, when applied in human diet, cannot be used as an energy source but rather as additives and supplements, which can increase the nutritional value and health beneficial effects of existing food products. The biosynthetic potential of both types, undifferentiated and differentiated plant in vitro systems, can be exploited to supply the target amounts of plant cell biomass with the desired phytochemical pattern (Figure 1). Here, monitoring of genetic stability, metabolite profiling and omics approaches (transcriptomics, proteomics and genomics) are essential for the selection of high‐producing lines. Once the production lines have been selected, the next step is optimization of the process conditions [12, 13, 14]. Different mathematical approaches, including factorial design, response surface methodology, and the newly developed artificial intelligence models, in combination with optimization algorithms can be applied to maximize the system productivity [15, 16, 17]. An additional increase in yields can be achieved by using elicitors, a specific type of light source, precursors feeding or modern immobilization strategies [5, 18‐24]. Some examples of recently commercialized food additives and cosmetic ingredients obtained by plant cell culture technology are presented in Table 1. A detailed review of more products could be found in recently published papers [1, 3, 4, 25].

**TABLE 1:** Some examples of innovative commercially available food additives and cosmetic ingredients obtained by plant cell culture technology

Product name	Plant	In vitro system	Application	Active compounds	Recommended use	Manufacturer	Web page
TEUPOL (10P or 50P)	*Ajuga reptans* L.	Cell suspension culture	Food additive	Teupolioside (standardized at 10% or 50 %)	Anti‐inflammatory; Modulates testosterone‐related disorders;	ABResearch srl	https://www.abres.it/teupolioside.php
					Has beneficial effect on benign prostatic hyperplasia;		
					Has beneficial effect on Crohn's syndrome;		
					Has beneficial effects on microvasculature diseases.		
ECHINAN 4P	*Echinacea angustifolia* DC.	Cell suspension culture	Food additive	Echinacoside (standardized at 4%)	Neuroprotective;	ABResearch srl	https://www.abres.it/echinacoside.php
					Anti‐inflammatory;		
					Anti‐neoplastic;		
					Anti‐aging;		
					Immunomodulatory;		
					Has beneficial effect on fighting flu and flu‐like infections;		
					Has beneficial effect on modulating immunosuppressive conditions;		
					Has beneficial effect on reducing risk of medical complications in upper respiratory tract infections;		
					Has beneficial effect on modulating the erythropoietin production without affecting red blood cells count;		
ACTEOS 10P	*Aloysia citrodora*	Cell suspension culture	Food additive	Verbascoside (standardized at 10%)	Anti‐hepatotoxic;	ABResearch srl	https://www.abres.it/verbascoside-acteoside.php
	Paláu.				Anti‐inflammatory;		
					Anti‐nociceptive;		
					Anti‐oxidant;		
					Anti‐hemolytic;		
					Reduce symptoms of arthritis;		
					Reduce symptoms of hypertension;		
					Reduce symptoms of Parkinson's disease;		
					Reduce symptoms of Alzheimer's disease;		
					Reduce symptoms of estrogenic mediated diseases;		
					Reduce symptoms of allergy type 1;		
					Reduce symptoms of intestinal mucosytis		
PhytoCellTec™ Symphytum	*Symphytum officinale* L.	Callus Culture	Cosmetics, skin care products	Water extract	Boost the regenerative power of epidermal stem cells and their ability to build new tissue;	Mibelle AG Biochemistry	https://mibellebiochemistry.com/phytocelltectm-symphytum
					Increase the thickness of the epidermis;		
					Improve barrier function.		
PhytoCellTec™ nunatak®	*Saponaria pumila* L.	Callus Culture	Cosmetics, skin care products	Water extract	Maintain dermal stem cell vitality after UV irradiation;	Mibelle AG Biochemistry	https://mibellebiochemistry.com/phytocelltectm-nunatakr
					Have anti‐roughness effect;		
					Improve skin density;		
					Improve skin elasticity and firmness		
DEOBIOME NONI	*Morinda citrifolia* L.	Plant stem cells	Cosmetics,	anti‐Quorum Sensing molecules; sugars	Protection and balance of microbiome;	Vytrus Biotech	https://www.vytrus.com/products/deobiome-noniprcf-the-biological-deodorant/?portfolioCats=17
			Deodorants,		Metabolism shift from lipids to polysaccharides;		
			Skin care products		Quorum Sensing inhibitors;		
					Skin health and detoxification;		
					Odor fingerprint modulation		
ARABIAN COTTON PRCF	*Gossypium herbaceum* L.	Callus culture	Cosmetics,	Phenols and flavonoids	Anti‐oxidant;	Vytrus Biotech	https://www.vytrus.com/products/arabian-cottonprcf-the-broad-spectrum-protector-against-photo-aging/?portfolioCats=17
			Skin care products,		Photoprotective;		
			Sun blocks and sun protectors		Enhance cell viability;		
					Modulate inflammatory response in Human Epidermal Progenitor Cells;		
					ECM boosting activity		
InnovaBioTech Haberlea	*Haberlea rhodopensis* Friv.	in vitro culture	Cosmetics,	Myconoside	Anti‐aging;	Innova BM	http://innovabm.com/portfolio-item/old-ship/
			Skin care products		Increase collagen and elastin synthesis;		
					Reduce UV‐induced dermis oxidation;		
					Increase skin elasticity;		
					Increase skin radiance		

### 2.1 Undifferentiated plant in vitro systems

Pluripotent cells in plants could be found in three major stem cell systems: shoot apical meristem, root apical meristem, and cambium. Interestingly, most of the differentiated plant cells are able to de‐differentiate and return to a proliferative pluripotent state [26]. This phenomenon is the basis underlying the strategy for development of in vitro cultivation platforms for mass production of undifferentiated plant cells [1, 2]. These expression platforms could be realized by propagation of de‐differentiated callus cultures, cell suspension of de‐differentiated plant cells and cell suspension of cambial meristem stem cells. Here it is important to note that most of the active cosmetic ingredients referred to as “Plant Stem Cells” are actually obtained by cultivation of de‐differentiated plant cells [3, 4]. There are only few products based on cultivation of cambial meristem stem cells [3, 4, 18, 25]. Although cambial meristem stem cells show fast uniform growth and high genetic stability, the level of secondary metabolites in cells remains low and stimulating strategies such as elicitation and two‐stage cultivation should be applied to increase the overall system productivity [18, 19]. Currently, most of the cosmetic ingredients obtained by plant cell culture technology available on the market are based on cultivation of de‐differentiated callus cultures or de‐differentiated plant cell suspension cultures [3, 4]. In vitro cultivation of plant cell suspensions in liquid medium can be easily scaled‐up in various bioreactor systems, whereas the propagation of callus cultures on solid or semi‐solid medium is a slow, costly and laborious process, which requires large production areas. It is noteworthy that in contrast to the rapidly growing number of commercialized active cosmetic ingredients, the progress in the development of food additives produced by cultivation of undifferentiated plant cell cultures is rather slow [13]. Recently, fresh and freeze‐dried cell cultures of three berry species (*Rubus* *chamaemorus* L., *Rubus saxatilis* L. and *Vaccinium vitis‐idaea* L.) have been evaluated for their nutritional values and potential use as food [27]. It was demonstrated that all cultures tested exhibit similar nutritionally valuable compounds, colors, visual appearance and sensory characteristics as those of the respective fresh berries. The authors reported that freeze‐dried cells have better digestibility properties and melt quickly in the mouth [27]. Recently, Teupol, released by ABResearch Srl, has become the first plant cell culture‐derived botanical ingredient approved in Europe as a food supplement (substantial equivalence). The company launched another two products, Echinan 4P and Acteos 10P, authorized as Novel Foods under the EU Regulation (Table 1).

### 2.2 Differentiated plant in vitro systems

Different plant tissues and organs can be isolated and cultivated under a controlled aseptic environment which supports their fast growth and maintains high levels of cell differentiation and tissue organization [6, 28, 29]. This involves in vitro cultivation of seedlings, stem cuttings, nodal segments, shoots, normal, adventitious or transformed “hairy” roots, somatic embryos, etc., widely known as differentiated plant in vitro systems. In contrast to undifferentiated plant in vitro systems, differentiated cultures are characterized by better genetic and biosynthetic stabilities and ability to produce and accumulate higher concentrations of certain specific metabolites whose biosynthesis occurs in specific plant tissues [6, 29‐31]. On the other hand, mass propagation of differentiated plant in vitro cultures is a costly and laborious process which requires specialized and expensive equipment [28, 32, 33]. Moreover, where the target products obtained by cultivation of differentiated plant in vitro cultures are intended for use as food or cosmetic ingredients, the fast growing transformed “hairy” root cultures are not recommended as a choice for production systems because of customers’ concerns about their “natural” status and safety [34]. Currently, there are only few examples of commercialized food or cosmetic ingredients obtained by cultivation of differentiated plant in vitro cultures [1, 3]. The most successful example is the large‐scale production (35 to 45 tons per year) of ginsenosides (triterpene saponins applied in functional foods and cosmetics) by cultivation of *Panax ginseng* adventitious root culture, which has been realized in South Korea by the CBN Biotech Company [34].

## 3 BIOREACTOR SYSTEMS USED FOR LARGE‐SCALE PRODUCTION OF FOODS AND COSMETIC INGREDIENTS BY PLANT CELL, TISSUE AND ORGAN CULTURES

To be cost‐effective, commercial production of plant cells or tissue biomass requires scale‐up of the process to the necessary volumes which can ensure a continuous supply. In this aspect, various bioreactor systems modified for growing undifferentiated or differentiated plant in vitro cultures can be adopted [28, 31–33, 35]. It is important to note that if the products are intended to be used in cosmetics, cultivation could be performed in small or medium volume bioreactors, or even in a solid medium (in multiple containers) or in small volumes agitated liquid medium (in multiple shaking flasks), whereas, if foods are the target market, cultivation should be conducted in large volume vessels. For example, the red dye shikonin (a naphthoquinone compound used in foods and also as an ingredient for the popular cosmetic product “biolipstick”) was commercially produced by Mitsui Petrochemical Industries Ltd. by developing a two‐stage cultivation process of cell suspension culture of *Lithospermum erythrorhizon* Siebold & Zucc. in 200 L (growth medium) and 750 L (production medium) stirred air‐lift bioreactors [36, 37]. Another food additive, ginseng roots, was produced by CBN Biotech Company by cultivation of *Panax ginseng* adventitious root culture in 1000 L balloon type air‐lift bioreactors [15, 34]. In contrast, Mibelle Biochemistry, which was the first company to introduce plant stem cells as cosmetic active ingredients, used modified 50 and 100 L disposable wave‐bioreactors for producing their flagman PhytoCELLTECH Malus domestica [1, 38]. Obviously, different types of bioreactors need to be implemented depending on the type of in vitro system and the nature of the ingredient produced. However, for optimizing the initial investment made in the equipment, disposable bioreactors as well as unconventional decisions such as temporary immersion systems should be considered [14, 28, 31, 33–35, 38, 39].

## 4 DOWNSTREAM PROCESS AND PRODUCT FORMULATION

Safety is the most important step in the development of active ingredients when used in foods or cosmetics. Plant cell culture technology offers by default continuous production of contamination‐free plant biomass. However, it should be noted that the post‐processing of this biomass is different if the final product is a food or cosmetic ingredient. In cosmetics, even though most companies claim to have “plant stem cells” in their products, they actually produce extracts [1, 3, 40]. Extracts are acellular products obtained by different methods. It is crucial for the cosmetic industry that only safe and non‐hazardous solvents, preferably of known natural origin, be used for extraction. They can be rich in the target bioactive compound or group of compounds, or they can be a bulk mix of disrupted cells where the entire cell content is presented [1, 3]. On the other hand, when the product will be used as functional food or food additive, the texture is essential and in addition to acellular extracts, cellular products could be more desirable [41]. Several types of cellular food additives obtained by plant cell culture technology, immobilized cells in alginate or agarose beads, structured “patties,” structured and dried “crisps” have been proposed but are still far from commercialization [27, 41]. Recently, with the advancement of 3D printing technology, new horizons in the development of flesh‐like printed cellular products have been produced by using developed food‐inks containing live plant cells and alginate or pectin matrix [42, 43].

## 5 CONCLUDING REMARKS

In the last few years, plant cell culture technology has gained the attention of modern food and cosmetic industries. The growing interest is driven by the search for alternative sustainable sources of plant‐derived natural bioactive products with low ecological footprints. However, in contrast to the exponentially growing number of commercialized cosmetic ingredients, the plant cell culture products used as food supplements are still limited. Currently, there are many bioreactor designs available, which can ensure cost‐effective large‐scale production of both differentiated and undifferentiated plant in vitro systems. However, the development of high‐productive and genetically stable cell lines, their maintenance and optimization of the cultivation conditions remain the most expensive, laborious and risky tasks. On the other hand, registration, certification and compliance with the national legislative requirements are additional costly and time‐consuming procedures, which can slow down product realization. However, the advances in extraction procedures (including application of recently developed natural deep eutectic solvents (NADES) as green solvents) and 3D printing can open new horizons in the development of high quality food and cosmetic active ingredients based on in vitro cultivation of plant cells and tissue cultures.

## CONFLICT OF INTEREST

The authors have declared no conflicts of interest.

## Data Availability

Data sharing not applicable to this article as no datasets were generated or analyzed during the current study.
